# Meaningful community engagement and involvement in global health and research: ‘Changing mindsets with a million conversations’ in Sierra Leone

**DOI:** 10.7189/jogh.14.03019

**Published:** 2024-04-12

**Authors:** Lucy November, Mangenda Kamara, Philemon Kamara, Suzanne Thomas, Appiah M Kingsford, Andrew H Shennan, Jane Sandall, Cristina Fernandez Turienzo, Prince T Williams

**Affiliations:** 1 Department of Women and Children’s Health, Faculty of Life Sciences and Medicine, King’s College London, London, UK; 2 University of Sierra Leone, Freetown, Sierra Leone; 3 Lifeline Nehemiah Projects, Freetown, Sierra Leone; 4 Welbodi Partnership, Freetown, Sierra Leone

Empowering and enabling communities to have control over decisions that affect their lives and giving them a genuine voice, is essential to achieving transformational impact and health for all, especially for the most disadvantaged, vulnerable and marginalised communities [1]. Much learning has been shared in recent years from a number of programmes and clinical trials [2–4], and meaningful engagement and involvement of communities in the planning, implementation, evaluation, and dissemination process has become a core practice for changing lives and reducing health inequities and informing policy-making [5]. Guides and tools are available to facilitate partnership with communities through intentional and structured community engagement and involvement (CEI), including ensuring quality standards and indicators (i.e. engagement, empowerment, ownership, inclusion, communication, adaptability, capacity building) [6–8] but evidence for approaches to and impact of CEI in low-income contexts is sparse [9,10]. Communities are complex, dynamic, and heterogeneous social groupings which may have different or even competing ideas about what they need; all engagement requires a deep understanding of that community’s social determinants of health and intersecting experiences [6]. Thus, contextually grounded approaches are more likely to involve communities meaningfully if they build and maintain trusting relationships with openness towards new ways of respectful and positive power-shifting and sharing, valuing and including different knowledge bases and perspectives, and using more participatory methods [6,11].

This is the CEI approach used by Lifeline Nehemiah Projects (LNP) [12], a community-based grassroots organisation based in a poor suburb of Sierra Leone’s capital, Freetown. LNP has a long history of engaging and involving stakeholders as change agents in their communities. Originally set up in 1996 with the purpose of rebuilding the lives of child soldiers and other war-affected children, the same vision of equipping communities for sustainable development continues to expand. Recognising the damage that can be done to existing local traditional leadership structures by short-term programmes, LNP is determined to engage and build relationships over time within these traditional structures for communities to own the process for finding local solutions whilst challenging mindsets undermining their development. Some of LNP’s recent successes included CEI activities to relieve tensions between or within villages across the country, to conduct sensitisation campaigns during the Ebola outbreak, to support rebuilding after the 2018 floods, and to raise awareness of blood donation by leading a community-hospital partnership to recruit volunteers and make blood readily available in hospitals to save lives.

In this case study, we describe LNP’s CEI approach to their 2YoungLives [13], mentoring intervention for pregnant and parenting adolescents. 2YoungLives was developed by LNP in 2017 to combat the stigma and mitigate the social disadvantage associated with adolescent pregnancy in Sierra Leone. Often abandoned by families and exiled from school, the outcomes for these girls are poor; late or absent antenatal and delivery care, poor diet and lack of emotional, financial and social support can all too often lead to maternal and perinatal mortality or morbidity, as well as poor social and economic outcomes [14]. 2YoungLives seeks to address these disadvantages by training local women with a reputation for kindness and compassion to mentor pregnant adolescents. The mentors support girls in starting small businesses, reconciling with families, attending antenatal care, breastfeeding exclusively, parent positively, taking up postpartum contraception, and returning to school or learning a trade. A pilot cluster randomised controlled trial is currently under way to understand the feasibility of 2YoungLives in different communities and inform a future larger trial and scale-up [14].

When planning this trial, LNP devised a CEI strategy to pave the way for the acceptance and tailoring of 2YoungLives in intervention communities. It was crucial to consider contextual factors, understand how communities operated, and engage those who would bring out various perspectives. The team made three trips to each site. An initial trip for introductions and to pay respects to the community chief and other leaders. A second to allow the community-wide airing of concerns and sharing of local beliefs and potential barriers and facilitators to the acceptance of the intervention and identification of women with a passion for supporting vulnerable girls as potential mentors. And a third to recruit mentors in collaboration with community stakeholders. Listening, discussing, and connecting were imperative to building trusting relationships. In all sites, significant barriers were raised and discussed; some were religious, some were based on previous history with other non-governmental organisations, and some were rooted in interpretations of current government policy. However, because these trips were factored into the timescale and budgeting of the project, there was time and space to address these barriers in respectful ways appropriate to each community and to mitigate them.

For example, in one community, parents were reluctant to let their girls enrol into the mentoring scheme because of the unfulfilled promises of other non-governmental organisations. The LNP team brought some key stakeholders to Freetown to see LNP work and meet 2YoungLives alumni who were now working and living independently, having completed LNP’s vocational training, for example, as plumbers or electricians, and were supporting their families. They were so impressed that on their return, they called a community-wide meeting to share their experience and ensure that their community’s children would benefit in a similar way. In another community, mentees were reluctant to attend the government peripheral health unit for antenatal visits for fear of a policy of reporting pregnant under 18 to the police to enforce identification and subsequent arrest of their baby’s father. This led to girls not attending or overstating their ages to avoid this risk. The LNP team engaged the peripheral health unit staff to discuss and understand this barrier to maternity care for this vulnerable group and invited them to the monthly 2YoungLives meeting to meet the mentees in a non-threatening environment. This rebuilt confidence in the PHU and reassured mentees that they would not be arrested for presenting to maternity care. Asking ‘How will this work in your community?’ and being ready to be flexible to respect, value and tailor approaches to different communities have been keys to success.

Previously, 2YoungLives was seen primarily as an intervention that positively affected individual adolescent girls. However, another layer of impact is emerging from qualitative data, which can be directly linked to LNP’s initial CEI strategy; that of whole community mindset change. There is a new awareness within the communities about the needs of pregnant teenagers; it is now acceptable and promoted for pregnant girls to go to school in accordance with the government’s radical inclusion policy, and even girls who are not currently mentored due to capacity limitations are being viewed differently by other community stakeholders since these difficult and sensitive topics have been aired openly in their communities.

There is a saying at LNP – ‘changing mindsets takes a million conversations’. These conversations need to be very respectful and take time, energy, and patience. However, if the result is shifting attitudes and mindsets to benefit young, marginalised groups, including girls and women, they are a hugely valuable investment.
